# Development of the thin film solid phase microextraction (TF-SPME) method for metabolomics profiling of steroidal hormones from urine samples using LC-QTOF/MS

**DOI:** 10.3389/fmolb.2023.1074263

**Published:** 2023-03-06

**Authors:** Wiktoria Struck-Lewicka, Beata Karpińska, Wojciech Rodzaj, Antoni Nasal, Bartosz Wielgomas, Michał Jan Markuszewski, Danuta Siluk

**Affiliations:** ^1^ Department of Biopharmaceutics and Pharmacodynamics, Medical University of Gdańsk, Gdańsk, Poland; ^2^ Department of Toxicology, Medical University of Gdańsk, Gdańsk, Poland

**Keywords:** thin-film solid phase microextraction TF-SPME, LC-QTOF/MS, endogenous steroid hormones, urine, untargeted metabolomics profiling

## Abstract

In the present study, the development and optimization of a thin film solid phase microextraction method (TF-SPME) was conducted for metabolomics profiling of eight steroid compounds (androsterone, dihydrotestosterone, dihydroepiandrosterone, estradiol, hydroxyprogesterone, pregnenolone, progesterone and testosterone) from urine samples. For optimization of extraction method, two extraction sorbents (PAN-C18 and PS-DVB) were used as they are known to be effective for isolation of low-polarity analytes. The stages of sample extraction and analyte desorption were considered as the most crucial steps in the process. Regarding the selection of the most suitable desorption solution, six different mixtures were analyzed. As a result, the mixture of ACN: MeOH (1:1, *v/v*) was chosen in terms of the highest analytes’ abundances that were achieved using the chosen solvent. Besides other factors were examined such as the volume of desorption solvent and the time of both extraction and desorption processes. The analytical determination was carried out using the ultra-high performance liquid chromatography coupled with high resolution tandem mass spectrometry detection in electrospray ionization and positive polarity in a scan mode (UHPLC-ESI-QTOF/MS). The developed and optimized TF-SPME method was validated in terms of such parameters as extraction efficiency, recovery as well as matrix effect. As a result, the extraction efficiency and recovery were in a range from 79.3% to 99.2% and from 88.8% to 111.8%, respectively. Matrix effect, calculated as coefficient of variation was less than 15% and was in a range from 1.4% to 11.1%. The values of both validation parameters (recovery and matrix effect) were acceptable in terms of EMA criteria. The proposed TF-SPME method was used successfully for isolation of steroids hormones from pooled urine samples before and after enzymatic hydrolysis of analytes.

## 1 Introduction

Steroid hormones are a group of hormones possessing in their chemical structure a sterane skeleton (Pawłowski, 2020). They could be divided in two classes: corticosteroids and sex steroids (Zeelen, 1997). Within those two classes are five types according to the receptors engaged in their pharmacological action: glucocorticoids, mineralocorticoids, estrogens, progestins and androgens (Brunton et al., 2011). The natural steroid hormones are generally synthesized from cholesterol in adrenal cortex (gluco- and mineralocorticoids) and in the gonads (estrogens, progestins and androgens) (Miller, 1988). They bind to specific serum carrier proteins (e.g., globulins) and are transported through the bloodstream to various target organs. Prior to their pharmacological effects presentation, steroids are liberated from carrier proteins, and, due to their high lipophilicity they easily pass the cell membrane, and are translocated to the nucleus where they bind to nuclear receptors. In the nucleus, the steroid-receptor ligand complex binds to specific DNA sequences and induces transcription of its target genes (Gupta and Lalchhandama, 2002; Rousseau, 2012).

Steroid hormones carry out regulation of such physiological processes as e.g*.*, carbohydrate, protein and fat metabolism (glucocorticoids) (Mantha et al., 1999; Kuo et al., 2015), and water-mineral balance (mineralocorticoids) (Rogerson and Fuller, 2000). Steroid hormones are also responsible for sexual differentiation and reproduction (sex steroids, gonadocorticoids). Estrogens, including estradiol, take part in the development of the primary and secondary female sex characteristics, while progestins (e.g., progesterone) in maintaining pregnancy. Androgens (e.g., testosterone) control the development and maintenance of reproductive function and are responsible for the secondary sex characteristics in the male (Brunton et al., 2011).

Solid phase microextraction method is a modern analytical sample pretreatment approach that allows for efficient isolation, matrix purification and analytes’ concentration in one process. SPME can be easily automated and requires low amounts of chemical solvents. It can be used for isolation of compounds of various physico-chemical properties and from variety of matrices (biological, environmental samples and others) (Pawliszyn, 2009). Therefore, it can also be adopted in various metabolomics studies (Vuckovic and Pawliszyn, 2011; Bojko et al., 2014; Jaroch et al., 2019; Mousavi et al., 2019). SPME process is composed of only a few steps that allows for matrix purification and analytes concentration which decreases the risk of possible contaminations, analytical errors or loss of analytes. Depending of the type of sorbent used, the SPME can be more or less selective in terms of the range of metabolome coverage from extracted samples (Olcer et al., 2019).

There are various types of SPME techniques among which the thin-film one (TF-SMPE) is of interest. Compared to the traditional microextraction techniques, the most important advantage of TF-SPME is that a larger volume of extraction phase (the so-called thin-film geometry) which is reflected as larger surface area, leads to higher extraction efficiency and enhanced sensitivity of extraction process. This approach provides more effective agitation as well as increased extraction recovery with shortened analysis time. When combined with automatic 96-blade system, TF-SPME provides high-throughput sample preparation involving preconditioning, sample extraction, washing, and sample desorption as automation process (Mirnaghi et al., 2011). Following Equation 1 (Eq. 1), the mass balance under equilibrium conditions, which is fundamental to SPME, presents the correlation between the amount of analytes extracted into the extractive phase (n) and its original concentration in the extracted sample (C_0_). According to Eq. 1, increase in the volume of extractive phase will lead to the enhancement of extraction sensitivity (Olcer et al., 2019).

$$n=\frac{K*V_{1}*V_{2}}{K*V_{2}+V_{1}}C_{0}$$ (1)

Where, *n*—number of moles of extracted compound, *K*- the distribution constant of the analyte for the extractive phase and sample matrix, *V*_*1*_- volume of sample, *V*_*2*_—volume of extractive phase, *C*_*0*_- original concentration of compound in sample.

The sensitivity enhancement is crucial in terms of extraction of trace amounts of compounds or when there is a need for extraction of compounds at different concentration levels from complex biological matrices. Therefore, some of the untargeted metabolomics studies have utilized TF-SPME for extraction and effective isolation of endogenous metabolites (Maciążek-Jurczyk et al., 2020; Łuczykowski et al., 2021). The purpose of the so-called metabolomic fingerprinting approach is to detect possibly all metabolites present in analysed sample. Hence, the sample pretreatment procedure is often limited only to dilution, deprotenization (in case of plasma), homogenisation (in terms of tissues) and filtration steps. The efficiency of matrix purification in SPME technique makes it an exceptionally useful analytical tool, however, some metabolites not absorbed to the SPME sorbent will not be ultimately detected. Therefore, this procedure can be used in metabolomics profiling approach which also belongs to the untargeted study but is focused on a selected group of metabolites with common chemical properties like semi-polar, non-polar metabolites or which belong to the same biochemical class such as nucleosides, steroid hormones, amino acids, fatty acids and others. Taking into account metabolomics profiling approach, the most crucial would be the selection of a proper SPME sorbent, dedicated to the metabolites of interests, as well the type of desorption solvent that can effectively desorb the compounds from the extractive phase. In regard with determination of steroid hormones, these analytes have been extracted using different modifications of SPME from various matrices like waste water, fish plasma, urine, milk, saliva or fish and chicken meat (Peñalver et al., 2002; Zhang et al., 2009; Kataoka et al., 2013; Lima Gomes et al., 2013; Olędzka et al., 2017; do Carmo et al., 2019; Mirzajani et al., 2019; Maciążek-Jurczyk et al., 2020; Wang et al., 2020). Although many SPME studies present the extraction of steroid hormones, only few adopting Thin-Film type of SPME have been found. In the work of Maciążek-Jurczyk et al. six steroid hormones (cortisol, testosterone, progesterone, estrone, 17β-estradiol and 17α-ethinylestradiol) were extracted using TF-SPME on C18 fibers from sucker fish plasma (Maciążek-Jurczyk et al., 2020). TF-SPME was also applied in a work of do Carmo et al. (do Carmo et al., 2019) for extraction of estrogens (estriol, estrone, 17β-estradiol and 17α-ethinylestradiol) from urine samples using specially synthetized biosorbent (bract) produced by the conifer *Araucaria angustifolia*. The determination was performed with the use of LC-FLD determination technique. Any other TF-SPME approaches have not been carried out for analysis of endogenous steroid hormones from human urine samples. For the first time in the present study, the polystyrene divinylbenzene (PS-DVB) as a thin-film extraction sorbent was chosen for isolation of steroid hormones from human urine samples. Steroid hormones from human urine were also extracted by Olędzka et al. (Olędzka et al., 2017) but the Authors utilized dispersive liquid-liquid microextraction which appeared to be more efficient than tested conventional type of SPME. Other SPME approaches for extraction of steroid hormones from pig urine (Zhang et al., 2009) river water (Lima Gomes et al., 2013), milk (Wang et al., 2020), white meat (Mirzajani et al., 2019) and saliva (Kataoka et al., 2013) were presented but those studies also utilized conventional SPME technique. Above all, the diversity of above mentioned approaches indicates that SPME is a prominent and modern analytical sample preparation technique which can be employed for effective isolation of endogenous compounds from matrices critical for bioanalytical or clinical studies. In an advent of Thin Film type of SPME, it revealed to be more robust and efficient extraction approach on even larger sample amount in relatively short time than conventional SPME.

In the present work, TF-SPME method was developed and optimized for extraction of eight steroid hormones (androsterone, dihydrotestosterone, dihydroepiandrosterone, estradiol, hydroxyprogesterone, pregnenolone, progesterone and testosterone). For optimization, the following types of parameters were tested: type of extraction phase, desorption solvent, time of both extraction and desorption processes as well as volume of the desorption solvent. The developed method was validated in terms of such parameters as the extraction efficiency, recovery as well as matrix effect according to the EMA regulations. The proposed TF-SPME method was applied for isolation of steroidal hormones from urine samples before and after enzymatic hydrolysis process.

## 2 Materials and methods

### 2.1 Chemicals, reagents and apparatus

The eight reference standards as estradiol, progesterone, androsterone, testosterone, dehydroepiandrosterone (DHEA), 17α-hydroxyprogesterone, 4,5α-dihydrotestosterone (DHT) were obtained from Merck, Darmstadt, Germany. Another reference standard pregnenolone was purchased from Avanti Polar Lipids, AL, United States. Deuterium labeled-d3-testosterone [d3-T] (100 μg/mL methanol solution) was purchased from Cerilliant Corporation (Austin, TX, United States). Methanol (MeOH), acetonitrile (ACN) and isopropanol (IPA), all of MS grade were purchased from Thermo Fisher Scientific (Massachusetts, United States). Deionized water was obtained using Milli Ro and Milli Qplus apparatus (Millipore, Vienna, Austria). Formic acid 98%–100% and glacial acetic acid (99%) of LC-MS grade, Surine™ negative urine control used as a blank urine were obtained from Supelco (Merck, Darmstadt, Germany). Sodium acetate trihydrate and β-glucuronidase from *Helix pomatia* (Type HP-2, aqueous solution, ≥100,000 units/mL), phosphate buffer saline (PBS) were also obtained from Supelco (Merck, Darmstadt, Germany). Reference mass solution and 10 times diluted ESI low calibration tuning mix were purchased from Agilent Technologies, Waldbronn, Germany). The SPME was performed using apparatus Concept 96.2 (PAS Technology, Magdala, Germany) composed of 96 well plates, the arm with mixing table and brush with blades coated in sorbent. The bladed brush was made from steel while the coatings were purchased from PAS Technology (Magdala, Germany). Extraction was performed by using steel blades coated with a polystyrene divinylbenzene (PS-DVB) and polyacrylonitrile C18 (PAN-C18) sorbents. Coating preparation procedures were based on the spraying method described by Mirnaghi et al. (Mirnaghi et al., 2011). The analyses were performed with the use of ultrahigh performance liquid chromatography UHPLC 1290 Infinity II Series (Agilent Technologies, Waldbronn, Germany) coupled with electrospray ionization (ESI) and high resolution tandem mass spectrometry 6546 QTOF/MS (Agilent Technologies, Waldbronn, Germany). The analyses were performed with the use of Mass Hunter Acquisition software whereas the obtained data were monitored and integrated using Mass Hunter Qualitative Analysis B.07.00 and Mass Hunter Profinder B.10.0 (Agilent Technologies, Waldbronn, Germany).

### 2.2 Chromatographic conditions

Analyses of estradiol, progesterone, androsterone, testosterone, DHEA, DHT, 17α-hydroxyprogesterone, pregnenolone and d3-testosterone were accomplished with the use of ZORBAX Extend C18 chromatographic column (2.1 mm × 100 mm, 3.5 μm; Agilent Technologies, Waldbronn, Germany). The mobile phase was composed of 0.1% aqueous solution of formic acid (phase A) and 0.1% formic acid solution in methanol (phase B). The gradient elution was utilized starting from 60% of phase B to 80% of B in 10 min, then was set at 80% of B for 4 min. The time for stationary phase equilibration was set at 6 min. The flow rate was 0.35 mL/min, the injection volume was 2 µL and the column temperature was maintained at 40°C.

### 2.3 Optimization of the mass spectra (MS) parameters

Mass spectra were recorded using full scan in positive ion mode with a scan range from *m/z* 61 to 1,000 to cover all steroid hormones likely to be detected. The analyses were performed using electrospray ionization source (ESI) with the following optimized parameters: gas temperature (nitrogen) was set at 320°C with flow rate at 10 L/min, nebulizer pressure was set to 40 psi, sheath gas temperature and its flow rate were set at 350°C and 11 L/min, respectively. The capillary voltage was maintained at 3250 V and fragmentor voltage was 150 V. The data were collected as centroids.

### 2.4 Preparation of standard stock solutions

The concentrated stock solutions of steroid hormones were prepared at 1 mg/mL in methanol. The working solutions of standards were prepared by dilution of stock solutions with methanol to obtain the following concentrations: 100 μg/mL, 10 μg/mL and 1 μg/mL. Standards at 1 μg/mL concentration level were analyzed separately using UHPLC- ESI-QTOF/MS in a scan mode to evaluate their retention time, ionization adducts and isotopic pattern.

For the development of TF-SPME method another working standard solution was prepared by mixing proper amount of each 100 μg/mL standard and methanol to the final concertation of 10 μg/mL. Such a mixture was diluted with 1% of PBS (1:10, *v/v*) to give the concentration of 1 μg/mL. The stock solutions were stored at −80°C while working standard solutions were kept in −20°C. Proper volume of each working solution was added to urine blank matrix (Surine™ negative urine control) in order to prepare quality control samples (QC) during validation process of SPME extraction.

### 2.5 SPME procedure

The developed and optimized SPME procedure was composed of five steps: preconditioning, extraction, washing, desorption and cleaning of sorbents. Each step was performed at room temperature at 1,000 rpm agitation speed. The extraction sorbent was preconditioned with 1 mL of methanol/water (50:50, *v/v*) for 30 min. Then 1 mL of sample was extracted for 30 min. After this step the blades were washed with deionized water for 10 s and subsequently, the desorption was applied with the mixture of methanol/acetonitrile (50:50, *v/v*) for 45 min. The samples after desorption phase were evaporated to dryness using vacuum centrifuge at 45°C. The dry residues were dissolved with 200 µL of methanol, centrifuged at 140,000 rpm for 10 min and injected into UHPLC-QTOF/MS system. After desorption, the sorbents were cleaned with the use of the mixture composed of methanol, acetonitrile, isopropanol and water (25:25:25:25,*v/v/v/v*). All extraction steps were performed at room temperature with 1,000 rpm agitation.

The optimization of TF-SPME relied on the evaluation of i) type of extraction sorbent, ii) type of desorption mixture, iii) time of both extraction and desorption processes and iv) volume of desorption mixture. The optimization of TF-SPME method was carried out with the use of mixture of standards as it was mentioned in 2.4. Section, wherein the 100 µL of mixture at concentration of 10 μg/mL spiked with internal standard (10 μL at 10 μg/mL), was dissolved with 900 µL of 1% PBS. Such 1 mL of extraction solvent was transferred to 96 well plates.

**FIGURE 1:**
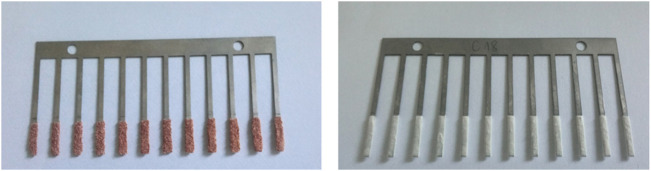
Two types of extraction solid phases used in the study. A-polystyrene divinylbenzene (PS-DVB) and B-polyacrylonitrile C18 (PAN-C18) sorbents.

The exemplary bladed brush coated with two extraction TF sorbents was presented in Figure 1.

The validation of TF-SPME was performed with the use of urine samples, wherein the 500 µL of urine spiked with internal standard (10 μL, 1 μg/mL) was diluted with 500 µL of 1% PBS. The application of TF-SPME for pooled urine samples was performed following the enzymatic hydrolysis reaction using a modified method described by Klimowska et al. (Klimowska and Wielgomas, 2018).

### 2.6 Preparation of urine samples and enzymatic hydrolysis procedure using β-glucuronidase from *Helix pomatia*

Steroid hormones which are excreted into urine are mainly their glucuronic or sulphate conjugates as their undergo metabolic II phase biotransformation. In order to detect unconjugated forms of steroids, those hormones in a urine sample should be enzymatically hydrolyzed. In the present work, the enzymatic hydrolysis was utilized with the use of β-glucuronidase obtained from *H. pomatia* (Type HP-2, activity ≥100,000 units/mL). The procedure was applied for the pooled urine obtained from healthy volunteers (n = 6). The pooled urine samples were derived from three women and three men (mean age: 41.67 ± 5.32, BMI: 22.43 ± 2.35). Prior to this study, an ethical approval from an independent committee of bioethical research at the Medical University of Gdansk was obtained (number of consent: NKBBN/252/2014). The group of healthy volunteers have declared a good health status and did not undergo any medical treatment at the time of urine collection. The collected and pooled urine samples were immediately frozen and stored at −80°C. In each case, before SPME extraction procedure, the urine samples were thawed at room temperature. Then the urine was adjusted to pH = 5 using 1M of acetate buffer. Next, the 500 µL of centrifuged urine, spiked with 10 µL of internal standard (1 μg/mL) was hydrolyzed with 5 µL of β-glucuronidase during 8 h at 37°C. Then, the reaction was stopped by rapidly cooling samples in ice. The 500 µL of urine diluted with 500 µL of 1% PBS was used for the next step of the TF-SPME procedure.

### 2.7 Validation of SPME method

#### 2.7.1 Matrix effect, recovery and process efficiency

Matrix effect (ME), recovery (RE) and process efficiency (PE) were performed with the use of quality control standards which were set at three concentration levels (LQC, MQC and HQC) using steroid-free urine matrix. Three sets of samples were prepared as follows: Set A was composed of a set of steroid-free urine matrix extracted by SPME. Then the extract was evaporated to dryness and the dry residue was dissolved in methanol and subsequently spiked with QC standards. Such samples were then injected into the UHPLC-QTOF/MS system. Set B consisted of a set of steroid-free urine matrix spiked with QC standards and then extracted by SPME. After extraction, the samples were evaporated to dryness and the dry residue of each sample was dissolved in methanol and then injected into UHPLC-QTOF/MS system. Set C was a set of neat QC standards dissolved in methanol injected into the UHPLC-QTOF/MS system. In each set the area under the peak of each analyte *versus* area under the peak of internal standard was measured (EMA, 2011).

The recovery (RE) was calculated by dividing obtained results from set A by the set B using Equation 2.

$$RE=\frac{SET\;B}{SET\;A}\;x\;100\;\%$$ (2)

The process efficiency (PE) was calculated by dividing the results obtained from set B by the set C using the Equation 3.

$$PE=\frac{SET\;B}{SET\;C}\;x\;100\;\%$$ (3)

The matrix effect (ME) was obtained by calculating matrix factor which is the result of dividing set A *versus* set C using the Equation 4.

$$MF=\frac{SET\;A}{SET\;C}$$ (4)

Next, the average value of MF and standard deviation of MF was calculated thanks to which the matrix effect could be expressed as coefficient of variation. This was obtained using the following equation:

$$ME=\frac{MF\;standard\;deviation}{MF\;average}\;x\;100\;\%$$ (5)

## 3 Results and discussion

### 3.1 Chromatographic and mass spectra conditions

**FIGURE 2:**
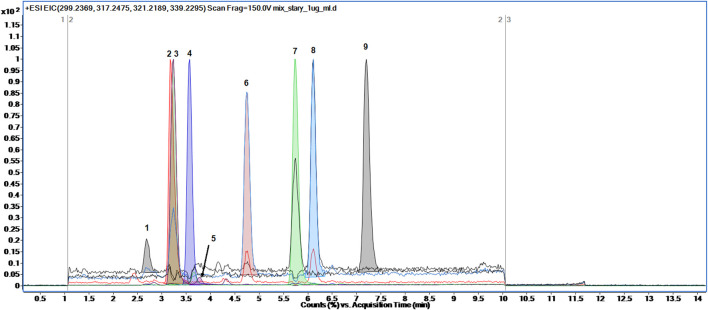
The representative Total Ion Chromatogram (TIC) of eight steroid hormones along with internal standard detected in full scan mode: one- estradiol, 2-d3-testosterone, 3-testosterone, 4-17α-hydroxyprogesterone, 5-DHEA, 6-DHT, 7-progesterone, 8-androsterone, 9-pregnenolone.

The analyses of eight steroid hormones (estradiol, progesterone, androsterone, testosterone, DHEA, DHT, 17α-hydroxyprogesterone, pregnenolone) along with the internal standard d3-testosterone was accomplished in 14 min using gradient elution composed of 0.1% FA in water and 0.1% FA in methanol according to the gradient program briefly presented in 2.2. Section. The retention time and ionization adducts of each analyte was measured by separate analysis of each steroid hormone in a full scan range from 61 to 1,000 m*/z*. The exemplary total ion chromatogram (TIC) of steroid hormones mixtures was presented in Figure 2. The peaks of analytes were extracted using Find Compounds by Formula algorithm in Mass Hunter Qualitative Analysis software.

Although the last detected steroid hormone eluted at 7.2 min the method lasted 14 min. The gradient elution set from 60% to 80% of organic modifier (phase B) was achieved from 0 to 10 min and then 80% of B was stated till 14 min. Such elution was developed in order to apply this method for separation of other steroid-related hormones in untargeted metabolomics profiling approach. Taking into account the structure of steroid hormones and their derivates possibly detected in urine samples, the gradient elution set to 80% of B seems to be enough to chromatographically separate other steroid-related compounds (Stanczyk et al., 1997; Boyaci et al., 2016).

Regarding ionization efficiency, it was observed that some steroid hormones appeared to have higher intensity when ionized with the loss of water molecule rather than by only protonation process. Therefore, such steroid hormones like estradiol, DHEA, androsterone and pregnenolone were monitored as their protonated adducts along with the loss of one molecule of water. The rest steroid hormones were monitored as their protonated adducts. The monitored precursor ions along with their retention times were presented in Table 1. Besides, in Supplementary Material (Supplementary Figure S1) the mass spectra of each detected steroid hormone were presented.

**TABLE 1:** The characteristics of detected steroid hormones including their molecular formula, mass, retention time and *m/z* of monitored ion.

Analyte	Molecular formula	Retention time [min]	Molecular mass [amu]	Monitored ion (*m/z*)
Estradiol	C_18_H_24_O_2_	2.68	272.1776	255.1743 (M + H^+^-H_2_O)
D3-Testosterone (ISTD)	C_19_H_25_O_2_D_3_	3.19	291.2278	292.2394 (M + H^+^)
Testosterone	C_19_H_28_O_2_	3.23	288.2089	289.2167 (M + H^+^)
17α-hydroxyprogesterone	C_21_H_30_O_3_	3.56	330.2195	331.2273 (M + H^+^)
DHEA	C_19_H_28_O_2_	3.77	288.2089	271.2059 (M + H^+^-H_2_O)
DHT	C_19_H_30_O_2_	4.74	290.2246	291.2322 (M + H^+^)
Progesterone	C_21_H_30_O_2_	5.73	314.2246	315.2319 (M + H^+^)
Androsterone	C_19_H_30_O_2_	6.11	290.2246	273.2216 (M + H^+^-H_2_O)
Pregnenolone	C_21_H_32_O_2_	7.22	316.2402	299.2372 (M + H^+^-H_2_O)

### 3.2 Development and optimization of TF-SPME procedure

**FIGURE 3:**
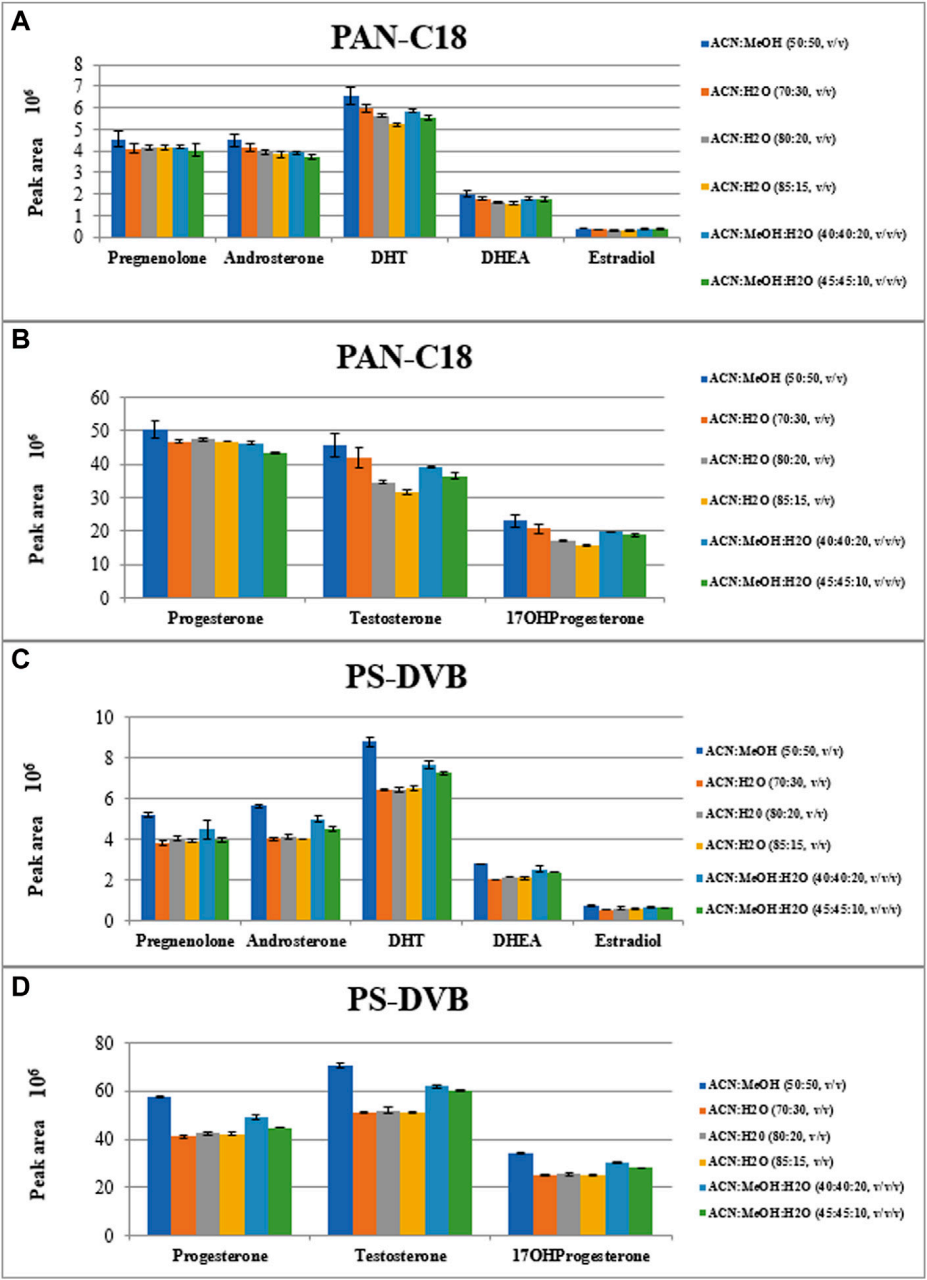
The peak areas of extracted steroid hormones depending on type of extraction sorbent **(A, B)**: PAN-C18; **(C, D)**: PS-DVB) and type of desorption mixtures.

The main objective of the present study was to develop and optimize the thin-film solid phase microextraction procedure for isolation of eight steroid hormones from urine samples. These steroid hormones are only the example of the widespread application of TF-SPME that is environmentally friendly, fast (if automated) and solvent-saving procedure. The optimized conditions of TF-SPME was optimized on various types of steroid hormones taking into account extraction of other steroid hormones in untargeted metabolomics profiling approach. Due to the capacious extraction phase in thin-film type of SPME in comparison with classical one, the sensitivity enhancement of the method is observed. Besides, the time of extraction can be shortened without the risk of sensitivity reduction. Here, two extraction sorbents were tested, namely, with the use of polystyrene divinylbenzene (PS-DVB) and polyacrylonitrile C18 (PAN-C18) sorbents. Both of these sorbents can be used for steroid-related compounds due to their affinity to low polar and hydrophobic molecules (Boyaci et al., 2016). For these fibers, various types of desorption mixtures were evaluated such as a) ACN:H_2_O (70:30, *v/v*); b) ACN:H_2_O (80:20, *v/v*); c) ACN:H_2_O (85:15, *v/v*); d) ACN:MeOH (50:50, *v/v*); e) ACN:MeOH:H_2_O (40:40:20, *v/v/v*) and f) ACN:MeOH:H_2_O (45:45:10, *v/v/v*). For the optimization of the type of sorbent and the desorption mixture, the 60 min extraction and 60 min desorption time was applied. The extraction was performed using three separate replicates of steroid hormones mixture. The results of TF-SPME extraction are presented in Figures 3A–D.

As it is observed in Figure 3, the most efficient desorption mixture was ACN:MeOH (50:50, *v/v*) where the peak area of each steroid hormone is the highest. Among sorbent types, the PS-DVB one, resulted in higher intensities of analytes than in the case of PAN-C18 sorbent. The exact values of peak area along with the coefficient of variation of the results are presented in Supplementary Materials Table S1.

**FIGURE 4:**
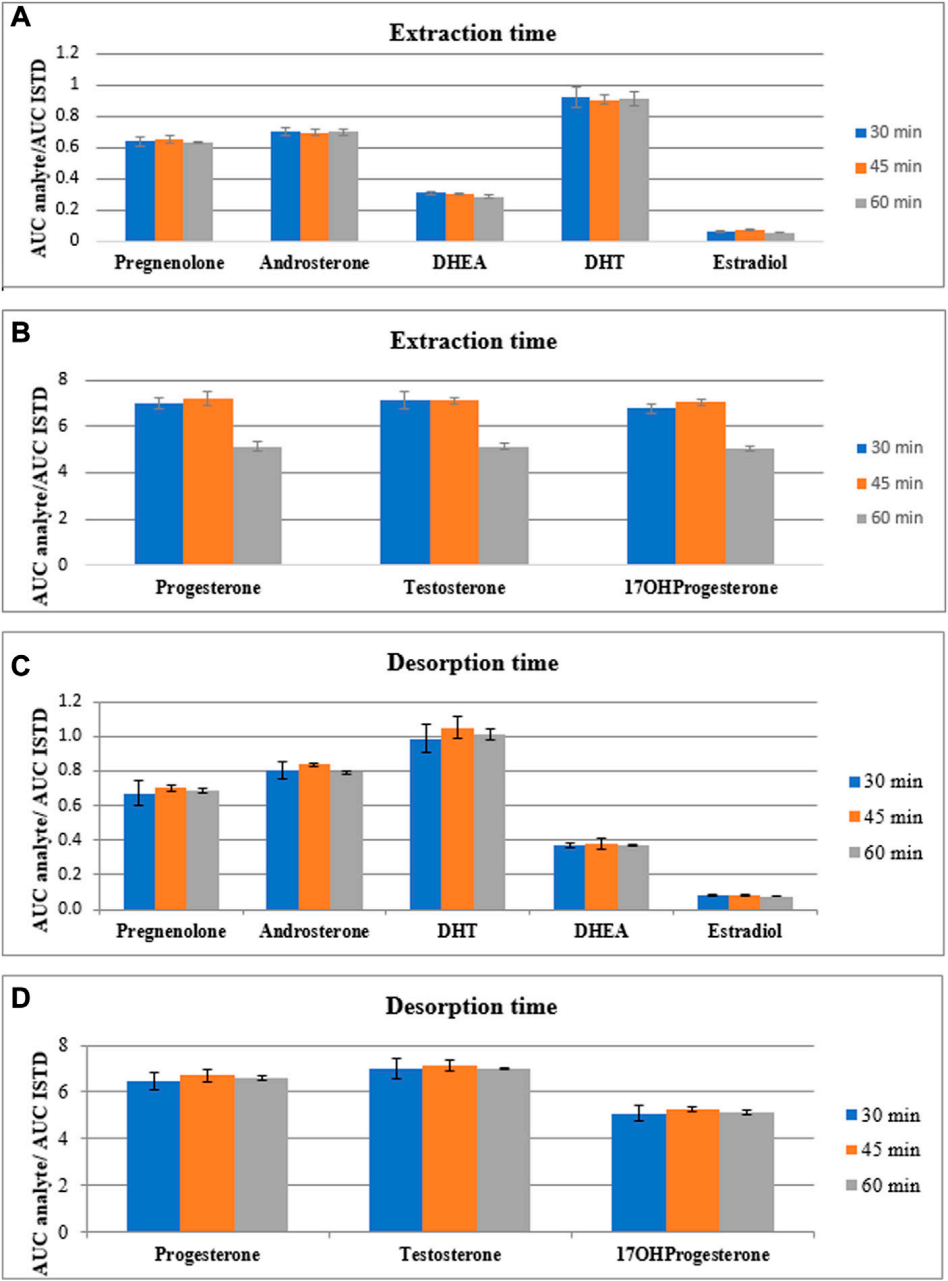
The comparison of peak area of steroid hormones normalized by peak area of internal standard for eight steroid hormones extracted **(A, B)** and desorbed **(C, D)** during various time points (30, 45 and 60 min).

After the selection of the fiber type and desorption mixture, the time of both extraction and desorption processes were evaluated. Due to the thin-film type of SPME, it is supposed that time of each step can be shortened in comparison with a classical SPME, as larger surface area leads to enhanced sensitivity of extraction process. The tested time of extraction and desorption processes were as follows: 30 min, 45 min and 60 min. Firstly, the time of extraction process was evaluated using 60 min desorption time. After final selection of extraction time, the time of desorption process was assessed. These experiments were carried out using four separate replicates and the results are presented in Figure 4. The exact values along with the coefficient of variations are presented in Supplementary Table S2, S3.

According to the obtained results related with the assessment of the extraction time, the intensities of analytes normalized by internal standard differ slightly and each time do not enhance significantly the sensitivity of the method. Also the coefficients of variation do not differ between time of extraction process. Therefore, taking into account the time of total SPME procedure, we decided to use 30 min extraction time for the next step of method development. Using this time of extraction, the time of desorption process was assessed. According to the results presented in Figure 4 and Supplementary Table S3, the higher intensities of the steroid hormones normalized by internal standard were observed during 45 min desorption time. The coefficient of variation was in acceptable range (<15%) but was varied between desorption time points. Due to the observed higher results obtained for 45 min desorption time, this time point for desorption step was chosen.

**FIGURE 5:**
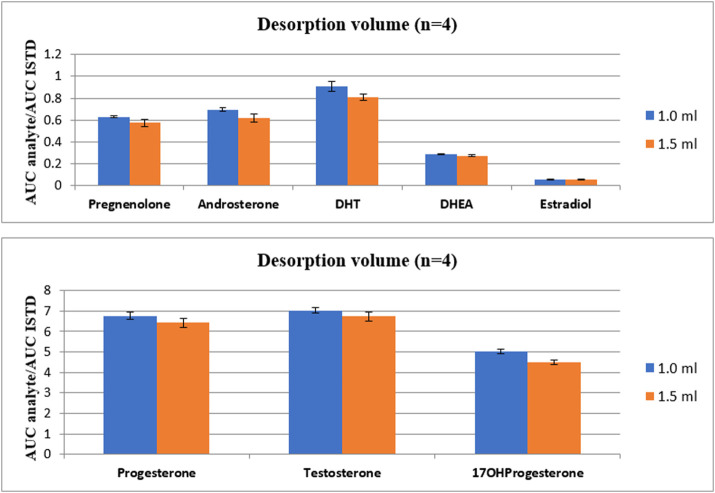
The comparison of the influence of desorption volume on the intensity of steroid hormones.

The last parameter evaluated in TF-SPME method was the volume of desorption. Here, two types of volumes were tested: 1 mL and 1.5 mL. The influence of applied two volumes of desorption mixture on analyte intensities is presented in Figure 5.

As it is presented in Figure 4, the lower desorption mixture volume applied, the higher intensity of analytes was observed. Therefore, the final volume of desorption mixture was set to 1 mL. It is important to emphasize, that the total time of TF-SPME procedure without cleaning step lasts less than 2 h. Taking into account 96 well plates present in the SPME apparatus, the extraction could be applied as high-throughput approach in untargeted metabolic profiling of steroid-related compounds.

The overall TF-SPME conditions applied for extraction and isolation of steroid hormones on DVB sorbent is presented in Table 2.

**TABLE 2:** Final conditions of developed and optimized TF-SPME method using PS-DVB sorbent for extraction of steroid-related compounds.

Name of the step	Type of solvent	Time [min]	Temperature [°C]	Agitation [rpm]
Conditioning	MeOH:H_2_O 1:1 (*v/v*)	30	20	1,000
Extraction	Sample	30	20	1,000
Washing	H_2_O	10 s	20	0
Desorption	ACN: MeOH 1:1 (*v/v*)	45	20	1,000
Cleaning	ACN: MeOH: IPA: H_2_O 1:1:1:1 (*v/v/v/v*)	60	20	1,000

### 3.3 Validation of the TF-SPME method

The developed and optimized method which final conditions were presented in Table 2 was validated in terms of recovery (RE), process (extraction) efficiency (PE) and matrix effect (ME). The calculations were performed using commercially available steroid-free urine matrix that was spiked with QC standards at three concentration levels (LQC = 0.25 μg/mL, MQC = 0.5 μg/mL and HQC = 0.75 μg/mL). The validation steps were briefly explained in 2.7.1. Section. The recovery and process efficiency were calculated using Eqs. 2, 3, respectively. For these values also percentage of standard deviation was calculated. Matrix effect was calculated using Eq. 4 where matrix factor was obtained. The coefficient of variation of matrix factor was calculated using Eq. 5. The results from validation are presented in Table 3. As it can be observed from the Table 3, the recovery and matrix effect are in acceptable range according to EMA validation criteria.

**TABLE 3:** Validation of developed TF-SPME method for extraction of steroid hormones from urine samples.

Analyte	Process efficiency			Recovery			Matrix effect [CV %]		
	LQC (n = 6)	MQC (n = 6)	HQC (n = 6)	LQC (n = 6)	MQC (n = 6)	HQC (n = 6)	LQC (n = 6)	MQC (n = 6)	HQC (n = 6)
Androsterone	88.0 ± 11.2	86.1 ± 7.9	81.9 ± 5.6	95.8 ± 3.4	95.3 ± 11.0	91.4 ± 6.5	9.1	2.8	3.4
DHEA	95.3 ± 4.8	93.1 ± 7.1	84.9 ± 7.2	106.8 ± 4.8	104.1 ± 8.3	96.0 ± 8.4	4.8	5.0	5.6
DHT	94.6 ± 9.8	91.3 ± 8.3	85.2 ± 6.0	102.8 ± 7.9	104.2 ± 10.7	98.0 ± 7.6	8.9	3.7	3.2
Estradiol	99.2 ± 3.6	90.9 ± 6.6	90.9 ± 4.1	107.1 ± 5.1	111.8 ± 8.1	100.2 ± 5.5	4.7	11.1	1.4
Pregnenolone	93.6 ± 9.6	98.7 ± 8.1	87.7 ± 7.9	102.1 ± 12	108.2 ± 10.5	96.3 ± 10.9	8.9	4.8	3.6
Progesterone	84.8 ± 7.3	83.3 ± 5.9	79.3 ± 4.5	89.9 ± 1.4	91.1 ± 8.4	88.8 ± 5.6	5.7	2.6	2.9
Testosterone	96.9 ± 6.1	91.3 ± 4.1	86.6 ± 2.5	106.2 ± 3.4	105.1 ± 7.5	99.3 ± 4.0	6.0	1.9	2.7
17αOHProgesterone	92.9 ± 8.5	90.8 ± 4.1	83.2 ± 3.5	101.6 ± 5.6	102.7 ± 8.5	93.8 ± 4.7	9.2	4.0	3.4
D3-Testosterone	85.4 ± 4.9	85.8 ± 4.3	87.8 ± 2.8	-----	-----	-----	-----	-----	-----

Table legend: LQC—low quality control, MQC—middle quality control, HQC—high quality control, CV—coefficient of variation.

### 3.4 Application of enzymatic hydrolysis of pooled urine samples using β-glucuronidase from *Helix pomatia*

As steroid hormones are excreted into urine mainly as glucuronic conjugates, their TF-SPME extraction of unconjugated forms of analytes from urine samples has to be performed after enzymatic hydrolysis. The hydrolysis was utilized using β-glucuronidase from *H. pomatia* with enzymatic activity ≥100,000 units/mL on pooled urine samples (n = 6) from healthy volunteers. β-Glucuronidase Type HP-2 from *H. pomatia* is a crude solution of enzymes derived from the digestive juices of the Roman snail. This type of enzyme (HP-2) has documented β-glucuronidase activity to be more than 100,000 units/mL as well as arylsulfatase activity at 7,500 units/mL level, therefore both glucuronic and sulfate conjugated of steroid hormones can be hydrolysed. The applied hydrolysis procedure was briefly presented in 2.6. Section. After hydrolysis, the pooled urine samples were extracted using validated TF-SPME method. To compare the influence of hydrolysis process on the analytes levels, the pooled urine samples without hydrolysis step were simultaneously extracted and analyzed. The determination was performed using developed and optimized method involving UHPLC-ESI-QTOF/MS instrumentation in a full scan mode. The extracted ion chromatograms (EIC) were prepared using find compounds by formula algorithm applied in Mass Hunter Qualitative Analysis B.07.00. Software. The change in levels of each steroid hormone was presented in Table 4.

**TABLE 4:** The influence of enzymatic hydrolysis on steroid hormones level using β-glucuronidase from *Helix pomatia*.

Analyte	Average area in pooled urine samples (n = 2)	Average area in pooled urine samples after enzymatic hydrolysis (n = 5)	Average change: Hydrolysis vs*.* without hydrolysis±SD	
Estradiol	ND	124046 (detected in 2 samples)	-----	
Testosterone	2374715	4474798	1.88	±0.06
17α-hydroxyprogesterone	3338358	693096	0.21	±0.1
DHEA	176344	17616511	99.9	±2.81
DHT	332358	1265096	3.81	±1.36
Progesterone	5839251	425985	0.07	±0.02
Androsterone	2210570	27497309	12.44	±0.67
Pregnenolone	149226	565234	3.79	±0.91

Table legend: SD—Standard deviation.

As it can be observed in Table 4, enzymatic hydrolysis significantly improved detection of DHEA. The level of DHEA after hydrolysis is almost 100 times higher than in comparison of its level without hydrolysis step. DHEA exists in urine also as sulphate conjugate but it is known that β-glucuronidases derived from molluscs often contain also sulfatase activity. The levels of such steroid hormones as testosterone, dihydrotestosterone, androsterone and pregnenolone were also from almost 2 to 4 times higher after enzymatic hydrolysis step. Concerning estradiol, this hormone was detected only in two out of five samples after hydrolysis so no comparisons were performed. The last hormones like progesterone and 17α-hydroxyprogesterone were found to have almost 14 and 5 times lower levels after enzymatic hydrolysis, respectively. The reason of that decreased level can be likely associated with another pathways of enzymatic biotransformation of these compounds. Progesterone can be metabolized to its main metabolite pregnanediol-3-glucuronide (PDG) (Stanczyk et al., 1997) so the balance between progesterone level itself can be moved to formation of other metabolites after enzymatic deconjugation like pregnanediol. Above all, the application of enzymatic hydrolysis step before the TF-SPME approach can be utilized in order to ensure better metabolome coverage in other untargeted metabolomics profiling studies.

### 3.5 Application of the TF-SPME method for untargeted metabolomics profiling studies

In the present study, the TF-SPME method was developed and validated based on eight steroid hormones from urine samples. However, taking into account the applied PS-DVB sorbent as well as type of desorption mixture (ACN:MeOH, 50:50, *v/v*) other steroid-related metabolites can be efficiently extracted as well. Additionally, the chromatographic parameters were optimized for determination of wider spectrum of metabolites, while the mass spectra conditions allow for detection of compounds in a very wide range of *m/z* from 61 to 1,000. In our previous study PS-DVB sorbent in TF-SPME was already applied for untargeted metabolomics study from urine samples, however, the method was not optimized for steroid hormones profiling (Łuczykowski et al., 2021).

In the present project the typical untargeted workflow has not been applied but the obtained set of data from pooled urine samples was processed using in-house database created based on Metlin Lipids library. Such database consisted of 43 steroid hormones and their derivatives. As a result, six additional steroids were annotated along with eight steroid hormones previously identified (based on reference standards). In Table 5, the list of additionally annotated steroid hormones is presented with the overall score of annotation set to be above 80%. The overall score includes match of isotopic pattern and molecular mass. Further studies in this untargeted steroid profiling are needed with the use of reference standards and MS/MS fragmentation pattern to confirm identity of additionally annotated compounds.

**TABLE 5:** Additional steroid hormones annotated from urine samples with the use of Find by Formula algorithm.

Name of analyte	Molecular formula	Molecular weight	*m/z*	Retention time [min]	Overall score
Deoxycortisol	C21H30O4	346.2153	347.2227	2.406	83.43
Dihydrocortisol	C21H32O5	364.2258	365.2326	2.422	86.15
Hydroxyandrosterone	C19H30O3	306.2199	289.2165	3.225	97.54
Tetrahydrocorticosterone	C21H34O4	350.2464	333.2429	3.39	88.41
Androsterone glucuronide	C25H38O8	466.2570	489.2463	4.027	98.21
Hydroxypregnenanolone	C21H34O3	334.2513	317.2482	5.616	98.24

## 4 Conclusion

The developed and validated TF-SPME method reported in this manuscript is simple, fast and with minimized influence of matrix effect on detection of steroid hormones in urine samples. The extraction method can be applied for isolation of steroid-related metabolites or other lipophilic compounds in untargeted/targeted metabolomics profiling approach. The utilized determination method involving UHPLC-ESI-QTOF/MS in a scan mode can be also applied for detection of urine samples to ensure the metabolome coverage of steroid related compounds.

## Data Availability

The original contributions presented in the study are included in the article/Supplementary Material, further inquiries can be directed to the corresponding author.
