# Supplementary material for: Development of the thin film solid phase microextraction (TF-SPME) method for metabolomics profiling of steroidal hormones from urine samples using LC-QTOF/MS
Source: Front Mol Biosci. 2023 Mar 6;10:1074263. doi: 10.3389/fmolb.2023.1074263 (PMC10025495; doi:10.3389/fmolb.2023.1074263)
Supplement: Supplementary file 1 [file Table1.DOCX]

**Supplementary materials:**

**Figure legends:**

**Fig. S1.** Mass spectra of detected steroid hormones according to which the monitored ion was chosen.

**Table legends:**

**Table S1**. Comparison of the average peak area of steroid hormones along with the coefficient of variation of the results for the selected desorption mixture ACN:MeOH (50:50, v/v) depending on the type of fiber used (PAN-C18 *vs* PS-DVB).

**Table S2.** Comparison of the average peak area of steroid hormones divided by area of internal standard along with the coefficient of variation of the results for the tested time of extraction process.

**Table S3.** Comparison of the average peak area of steroid hormones divided by area of internal standard along with the coefficient of variation of the results for the tested time of desorption process.

**Table S4.** The carryover effect after desorption steps and cleaning of DVB extraction phase used for isolation of selected steroid hormones.

Fig. S1. Mass spectra of detected steroid hormones according to which the monitored pseudomolecular ion was chosen.

Fig. S1 (*continued*). Mass spectra of detected steroid hormones according to which the monitored pseudomolecular ion was chosen.

**Table S1.** Comparison of the average peak area of steroid hormones along with the coefficient of variation of the results for the selected desorption mixture ACN:MeOH (50:50, v/v) depending on the type of fiber used (PAN-C18 *vs* PS-DVB).

| **Analytes** | **PAN-C18** | | **PS-DVB** | |
| --- | --- | --- | --- | --- |
|  | **Average (n=3)** | **CV [%]** | **Average (n=3)** | **CV [%]** |
| Androsterone | 4491281 | 7% | 5636123 | 1% |
| DHEA | 2011746 | 9% | 2775954 | 1% |
| DHT | 6568201 | 6% | 8781307 | 2% |
| Estradiol | 403077 | 5% | 757543 | 5% |
| Pregnenolone | 4562744 | 7% | 5221547 | 2% |
| Progesterone | 50408160 | 5% | 57547019 | 1% |
| Testosterone | 45734252 | 8% | 70751236 | 1% |
| 17OHProgesterone | 23056325 | 8% | 33959871 | 1% |

**Table S2.** Comparison of the average peak area of steroid hormones divided by area of internal standard along with the coefficient of variation of the results for the tested time of extraction process.

| **Time of extraction** | **30 min** | | **45 min** | | **60 min** | |
| --- | --- | --- | --- | --- | --- | --- |
| **Analytes** | **average**  **(n=4)** | **CV [%]** | **average**  **(n=4)** | **CV [%]** | **average**  **(n=4)** | **CV [%]** |
| Androsterone | 0.700 | 3.6% | 0.694 | 2.7% | 0.697 | 2.8% |
| DHEA | 0.308 | 3.1% | 0.300 | 2.3% | 0.287 | 2.3% |
| DHT | 0.921 | 6.9% | 0.904 | 3.4% | 0.911 | 4.8% |
| Estradiol | 0.063 | 8.6% | 0.070 | 7.7% | 0.057 | 2.8% |
| Pregnenolone | 0.639 | 4.7% | 0.651 | 3.6% | 0.630 | 0.8% |
| Progesterone | 6.986 | 3.4% | 7.130 | 4.8% | 6.769 | 2.7% |
| Testosterone | 7.204 | 4.2% | 7.108 | 1.8% | 7.032 | 2.1% |
| 17OHProgesterone | 5.136 | 3.5% | 5.129 | 2.3% | 5.024 | 2.2% |

**Table S3.** Comparison of the average peak area of steroid hormones divided by area of internal standard along with the coefficient of variation of the results for the tested time of desorption process.

| **Time of desorption** | **30 min** | | **45min** | | **60 min** | |
| --- | --- | --- | --- | --- | --- | --- |
| **Analytes** | **average (n=4)** | **CV[%]** | **average (n=4)** | **CV[%]** | **average (n=4)** | **CV[%]** |
| Androsterone | 0.805 | 6.2% | 0.836 | 1.5% | 0.793 | 1.0% |
| DHEA | 0.368 | 4.0% | 0.379 | 8.1% | 0.372 | 1.0% |
| DHT | 0.988 | 8.4% | 1.051 | 5.7% | 1.009 | 2.9% |
| Estradiol | 0.082 | 1.9% | 0.084 | 7.2% | 0.076 | 1.6% |
| Pregnenolone | 0.672 | 10.3% | 0.703 | 2.7% | 0.686 | 1.6% |
| Progesterone | 6.461 | 5.3% | 6.702 | 4.1% | 6.576 | 1.5% |
| Testosterone | 6.982 | 6.2% | 7.160 | 3.4% | 7.025 | 0.4% |
| 17OHProgesterone | 5.081 | 6.5% | 5.264 | 2.3% | 5.123 | 1.4% |

**Table S4.** The carryover effect after desorption steps and cleaning of DVB extraction phase used for isolation of selected steroid hormones.

| **Analyte** | **Mean area after 1^st^ desorption (n=3)** | **Mean area after 2^nd^ desorption**  **(n=3)** | **Carryover [%]** | **Mean area after cleaning (n=3)** |
| --- | --- | --- | --- | --- |
| Estradiol | 757543 | ND | --- | ND |
| Testosterone | 70751236 | 4279624 | 6.05 | ND |
| 17α-hydroxyprogesterone | 33959871 | 1602240 | 4.72 | ND |
| DHEA | 2775954 | 129611 | 4.67 | ND |
| DHT | 8781307 | 529877 | 6.03 | ND |
| Progesterone | 57547019 | 4100256 | 7.13 | ND |
| Androsterone | 5636123 | 288234 | 5.11 | ND |
| Pregnenolone | 5221547 | 369475 | 7.08 | ND |
